# Vitamin A supplementation boosts control of antibiotic-resistant *Salmonella* infection in malnourished mice

**DOI:** 10.1371/journal.pntd.0008737

**Published:** 2020-10-02

**Authors:** Annica R. Stull-Lane, Kristen L. Lokken-Toyli, Vladimir E. Diaz-Ochoa, Gregory T. Walker, Stephanie A. Cevallos, Andromeda L. N. Winter, Ariel Del Hoyo Muñoz, Guiyan G. Yang, Eric M. Velazquez, Chun-Yi Wu, Renée M. Tsolis

**Affiliations:** 1 Department of Microbiology & Immunology, School of Medicine, University of California Davis, Davis, California, United States of America; 2 Department of Microbiology, New York University, New York, New York, United States of America; 3 Department of Veterinary Clinical Sciences, College of Veterinary Medicine, China Agricultural University, Beijing, China; 4 Department of Neurology, School of Medicine, University of California, Davis, Sacramento, California, United States of America; University of Texas Medical Branch, UNITED STATES

## Abstract

Disseminated disease from non-typhoidal *Salmonella enterica* strains results in >20% mortality globally. Barriers to effective treatment include emerging multidrug resistance, antibiotic treatment failure, and risk factors such as malnutrition and related micronutrient deficiencies. Individuals in sub-Saharan Africa are disproportionately affected by non-typhoidal *S*. *enterica* bloodstream infections. To inform a clinical trial in people, we investigated vitamin A as a treatment in the context of antibiotic treatment failure in a mouse model of vitamin A deficiency. Vitamin A-deficient (VAD) mice exhibited higher systemic bacterial levels with a multidrug-resistant clinical isolate in comparison to mice on a control diet. Sex-specific differences in vitamin A deficiency and disseminated infection with *S*. *enterica* serotype Typhimurium (*S*. Typhimurium) were observed. VAD male mice had decreased weight gain compared to control male mice. Further, infected VAD male mice had significant weight loss and decreased survival during the course of infection. These differences were not apparent in female mice. In a model of disseminated *S*. Typhimurium infection and antibiotic treatment failure, we assessed the potential of two consecutive doses of vitamin A in alleviating infection in male and female mice on a VAD or control diet. We found that subtherapeutic antibiotic treatment synergized with vitamin A treatment in infected VAD male mice, significantly decreasing systemic bacterial levels, mitigating weight loss and improving survival. These results suggest that assessing vitamin A as a therapy during bacteremia in malnourished patients may lead to improved health outcomes in a subset of patients, especially in the context of antibiotic treatment failure.

## Author summary

Non-typhoidal *Salmonella* serotypes generally cause diarrhea in people. However, there are certain factors that make people at risk of developing a more severe infection where the bacteria can enter the blood and cause fever and whole-body symptoms. Patients with this infection are usually hospitalized, and about one in five patients do not survive. The factors that make this bloodstream infection possible include pathogen features like resistance to antibiotics and patient factors like a malnourished state. Better treatments are needed. In this study, the authors assess vitamin A as a treatment during antibiotic treatment failure in a mouse model. Vitamin A-deficient male mice have better outcomes with vitamin A and antibiotic co-therapy, whereas female mice do not benefit. Despite similar levels of bacteria causing infection systemically, female mice show better outcomes in terms of weight loss and survival than male mice overall. This research provides evidence that a clinical study assessing vitamin A as a treatment in people could lead to improved survival for malnourished patients presenting with severe bloodstream infection.

## Introduction

In susceptible populations globally, there is a vicious cycle of infection and malnutrition [1]. Malnutrition overlaps with deficiencies in micronutrients, such as iron, iodine, zinc and fat-soluble vitamins like vitamin A [2]. Severe or recurrent infections can lead to vitamin A deficiency due to impaired nutrient absorption and decreased food intake, as well as direct nutrient loss through urine [3, 4]. In turn, vitamin A deficiency compromises the gastrointestinal mucosal epithelial barrier, increasing susceptibility to enteroinvasive bacteria and sepsis [4]. One of the most common enteric pathogens is *Salmonella enterica* [5]. Non-typhoidal serotypes of *S*. *enterica* cause significant morbidity and mortality worldwide, resulting in 93.8 million cases of gastroenteritis and 155,000 associated deaths annually [6]. Invasive non-typhoidal *Salmonella* (iNTS) infection, a severe febrile illness with bacteremia, disproportionately affects infants, older adults and immunosuppressed individuals. An estimated 3.4 million cases of invasive disease occur globally per year, resulting in 680,000 deaths [7]. Malnutrition and concurrent malaria are key risk factors for systemic disease, and populations in sub-Saharan Africa are disproportionately affected [8]. Barriers to effective treatment of iNTS illness include appropriate diagnostic tools, antibiotic treatment failure and antimicrobial resistance [9]. Notably, the mortality rate due to iNTS in children reaches 20–25% [10], indicating that more effective treatment is vital.

Vitamin A administration has been studied as treatment of infectious disease, such as for measles in children [11]. In addition, the WHO recommends vitamin A supplementation in the treatment of children that present with severe acute malnutrition [12]. Given the importance of vitamin A in immune function [4, 13, 14], these treatment recommendations suggest a role for vitamin A as a host-directed therapy [15]. However, vitamin A has not been studied as a treatment for nonspecific febrile illnesses like iNTS disease, especially in the context of antibiotic treatment failure.

This study aimed to explore the therapeutic potential of vitamin A administered after onset of infection, simulating care of a sick patient presenting to clinic. Our hypothesis was that vitamin A therapy could boost the effect of subtherapeutic antibiotic treatment and improve treatment outcomes for a drug-resistant iNTS infection. To address this question, we used mice to model antibiotic treatment failure of *S*. *enterica* serotype Typhimurium in the setting of vitamin A deficiency.

## Materials and methods

### Ethics statement

Mouse experiments were carried out in compliance with the National Institutes of Health policies on animal welfare, the Animal Welfare Act, and all other applicable federal, state and local laws. Mice were cared for by research staff and University of California (UC) Davis Teaching and Research Animal Care Services (TRACS) under a program accredited by the Association for Assessment and Accreditation of Laboratory Animal Care International (AAALAC). TRACS maintains a health monitoring program administered through the UC Davis Comparative Pathology Laboratory. When needed, Campus Veterinary Services is available to provide interventional and supportive care. All mouse experiments were approved by the UC Davis Institutional Animal Care and Use Committee (IACUC) under protocol 19900.

### Animals

Inbred C57BL/6J-*Slc11a1*^+/+^ mice are a C57BL/6J congenic strain containing a genomic segment with *Slc11a1* from S29S1 mice that was introgressed into Chromosome 1 [16]. This mouse line was maintained at UC Davis under specific pathogen-free conditions in cages with sterile ALPHA-dri bedding manufactured by Shepherd Specialty Papers (Watertown, TN). To generate VAD and control mice, C57BL/6J-*Slc11a1*^+/+^ dams were given a VAD diet at 2 weeks of gestation. Pups were placed on either VAD or control diets at weaning and were maintained as groups throughout each experiment. Mouse weights were monitored weekly during growth. Custom VAD and control diets were obtained from Envigo Teklad Diets (Madison, WI). When the model was established, liver retinol concentration was confirmed by HPLC. Liver retinol concentration of control mice ranged from 120–300 nmol/g, whereas liver retinol concentration of VAD mice ranged from 0–7 nmol/g. Inbred C57BL/6J mice were purchased from The Jackson Laboratory (Bar Harbor, ME) and were maintained on standard chow.

### *In vitro* minimum inhibitory concentration experiment

D23580, a multidrug-resistant *Salmonella enterica* serotype Typhimurium clinical bloodstream isolate, was received from Robert Heyderman at the Malawi-Liverpool-Wellcome Trust Clinical Research Centre [17]. Inocula were cultured aerobically in Luria-Bertani (LB) media with 30 μg/ml chloramphenicol shaking for 16–18 hours at 37°C, subcultured to a calculated optical density (OD) of 0.001, and grown 16–18 hours rocking at 37°C in a 96-well plate containing LB with serial dilutions of enrofloxacin ranging from 0 μg/ml to 2.56 μg/ml. Enrofloxacin (Baytril 100 by Bayer) was obtained from the Pharmaceutical Service at the UC Davis Veterinary Medical Teaching Hospital. The OD was read at 595 nm on a BioRad Model 680 Microplate Reader (Hercules, CA). The experiment was repeated six times, and the average minimum inhibitory concentration (MIC) was recorded. A determination of susceptible (S), intermediate (I) or resistant (R) was made according to CLSI guidelines [18].

### *In vivo* infection with *Salmonella enterica* serotype Typhimurium

Inocula of *S*. Typhimurium strains D23580, a multilocus sequence type (ST) 313 strain isolated from blood, SL1344, an ST19 isolate from a calf with salmonellosis [19] and SARA16, an ST19 human isolate and reference strain [20, 21], were cultured in LB with shaking for 16–18 hours at 37°C. The identity of each strain was confirmed by antibiotic resistance profiling. Mice received 100 μl of sterile PBS or 100 μl of 1000 colony-forming units (CFU) diluted in sterile PBS by intraperitoneal (i.p.) injection. Mouse weights were monitored daily during infection. Mice were euthanized if they showed signs of lethal morbidity. Systemic bacterial levels were characterized at day 1, 4 or 5 post-infection by determining tissue loads of *S*. Typhimurium (CFU). Liver and spleen were collected in PBS, weighed and homogenized using an Ultra Turrax T25 Basic mixer from IKA (Staufen, Germany). Blood was collected in K2 EDTA microtainer tubes (Becton, Dickinson and Company, Franklin Lakes, NJ), kept on ice for one hour, and then incubated for 10 minutes with 100–200 μl of 1% Triton X-100. Liver, spleen and blood samples were serially diluted and plated on LB agar plates containing appropriate antibiotic selection. CFU/gram tissue or CFU/ml was calculated after overnight growth at 37°C.

### *In vivo* treatment experiments

Mice were infected with *S*. Typhimurium D23580 as previously described. For preliminary antibiotic treatment experiments, *S*. Typhimurium systemic bacterial levels (CFU) were assessed for male (n = 5) and female (n = 5) mice on a standard diet 5 days post-infection for the following treatment groups: 0 mg/ml, 0.01 mg/ml, 0.05 mg/ml, and 0.10 mg/ml enrofloxacin delivered in the drinking water. Water and mouse weights were collected daily, and the average antibiotic consumed (μg/kg/day) was determined. For co-treatment experiments with mice on special diets, 4–6 mice were assessed for control and VAD male and female mice in the following groups: mock-treated, mock-treated and enrofloxacin, vitamin A only, and vitamin A and enrofloxacin co-treatment. Mock-treated mice received 100 μl of sterile PBS administered by oral gavage on day 1 and day 2 post-infection. Vitamin A-treated mice received 600 IU of retinyl palmitate (Interplexus, Inc., Kent, WA) in 100 μl of sterile PBS administered by oral gavage on day 1 and day 2 post-infection and were changed to control diet at day 1 post-infection. Enrofloxacin (0.05 mg/ml or 0.01 mg/ml) was administered at day 2 post-infection in the drinking water. Enrofloxacin (Baytril 100 by Bayer) was obtained from the Pharmaceutical Service at the UC Davis Veterinary Medical Teaching Hospital. Water weights were monitored daily to determine average antibiotic consumption per mouse. CFU/gram tissue or CFU/ml was calculated for liver, spleen and blood. For measurement of plasma enrofloxacin concentrations, blood was first centrifuged for 10 minutes at 1000 x *g*. Plasma was stored at -80°C for further analysis by liquid chromatography with tandem mass spectrometry (LC-MS/MS). The remaining blood sample was incubated at room temperature for 10 minutes with 100–200 μl of 1% Triton X-100 and plated for CFU as previously described.

### Plasma antibiotic measurements

LC-MS/MS was used to quantify enrofloxacin and ciprofloxacin concentrations in the mouse plasma. First, a mixed master stock solution of 10 μg/ml of enrofloxacin (MilliporeSigma, St. Louis, MO) and ciprofloxacin (MilliporeSigma, St. Louis, MO) was made in control CD-1 mouse plasma (Bioreclamation IVT, Westbury, NY) with K2 EDTA. This stock solution was then further diluted with the same control mouse plasma to establish a mouse plasma calibration curve. The following calibrator concentrations were used: 0, 1, 2.5, 5, 10, 50, 100, 500, 1000 and 5000 ng/ml. Calibrators 10, 100 and 1000 ng/ml also served as quality control (QC) samples. Moxifloxacin (MilliporeSigma, St. Louis, MO) in acetonitrile (ACN) (Fisher Scientific, Hampton, NH) was used as an internal standard such that 160 μl of 200 ng/ml moxifloxacin in ACN was added to 40 μl of the mouse plasma calibrators, QC samples and the experimental mouse plasma samples collected as previously described. All samples were vortexed for 10 seconds. Samples were then centrifuged at room temperature for 5 minutes at 17,000 x *g*, and 25 μl of resulting supernatant was diluted with 175 μl of water. Next, 2 μl of the final solution was injected into the ACQUITY Ultra Performance Liquid Chromatography (UPLC) System with a BEH C18 column, 1.7 μm, 2.1 mm x 50 mm (Waters Corporation, Milford, MA). The following mobile phases were used: A (H_2_O with 0.1% formic acid), and B (ACN with 0.1% formic acid) (Fisher Scientific, Hampton, NH). Mobile phase A was also used for purging, and ACN/H_2_O at 50/50 (v/v) was used for the needle washing between each injection. The flow rate was 0.2 ml/min, the column temperature was set at 50°C and the autosampler temperature was set at 10°C. The following UPLC gradient program was used for the separation: 0–1 min, 10% B; 2.5 min, 72.5% B; 2.51–4.75 min, 95% B; 4.76–7 min, 10% B. The output of the UPLC was fed to a Xevo TQ-S Triple Quadrupole Mass Spectrometry (MS/MS) system (Waters Corporation, Milford, MA), which was used to ionize target molecules with electrospray ionization (ESI+) and monitor the ion m/z fragmentation transitions from 360.1–245.2 for enrofloxacin quantification, 332.2–245.2 for ciprofloxacin quantification and 402.2–261.1 for moxifloxacin quantification in multiple reaction monitoring (MRM) mode. The retention times were 2.45, 2.38 and 2.58 minutes for enrofloxacin, ciprofloxacin and moxifloxacin, respectively. The calibration curve was fitted with a weighted (1/x^2^) least-squares linear regression algorithm. The detection range was from 1 ng/ml to 5000 ng/ml for enrofloxacin and from 2.5 ng/ml to 5000 ng/ml for ciprofloxacin. The extraction yield was about 80–100% for all three antibiotics and the matrix effect enhanced both enrofloxacin and ciprofloxacin MS signals by 10% and 4%, respectively. The matrix effect enhanced moxifloxacin MS signals by 48%. Both inter- and intra-batch accuracy were lower than 10% (% deviation) except that ciprofloxacin had 11% of inter-batch deviation at 2.5 ng/ml. Both intra- and inter-batch precision were also lower than 10% coefficient of variation (CV), except that enrofloxacin and ciprofloxacin had 20–40% CV at 1 ng/ml and 2.5 ng/ml, respectively. The Lower Limit of Quantification (LLOQ) for enrofloxacin was determined to be 1 ng/ml and the LLOQ for ciprofloxacin was determined to be 2.5 ng/ml.

### Survival curves

To assess survival in untreated mice, control and VAD male and female mice were infected with *S*. Typhimurium D23580 as previously described, and weights were monitored daily for up to 4 days post-infection. Percent survival for each group (n = 8–9) was reported with a Kaplan-Meier survival curve. To assess male-specific survival with treatment conditions, control and VAD male mice were infected with *S*. Typhimurium as previously described. Control mice and one group of VAD mice were mock-treated with PBS. Other VAD groups received either enrofloxacin treatment alone (0.01 mg/ml) or co-treatment of enrofloxacin and vitamin A as previously described. Percent survival of 6–16 mice/group at 8 days post-infection was assessed with a Kaplan-Meier survival curve. To assess treatment of vitamin A alone, control and VAD male and female mice were infected with *S*. Typhimurium as previously described. Control mice were mock-treated with PBS, one group of VAD mice was treated with vitamin A as previously described and a second group of VAD mice received mock-treatment. Percent survival of 12 mice/group at 13 days post-infection was assessed with a Kaplan-Meier survival curve. For all survival curve experiments, mice were weighed daily and humanely euthanized when they approached 20% weight loss or displayed signs of lethal morbidity, according to our institution’s humane endpoints policy.

### Statistical analysis

Statistical analyses were performed with GraphPad Prism 8 (GraphPad, La Jolla, CA). Since data were found not to have a normal distribution, nonparametric analyses were utilized. Significance of differences between two groups was determined with a Mann-Whitney test. Significance of differences between multiple groups from a control group was determined with a Kruskal-Wallis test and a *post-hoc* Dunn’s multiple comparisons test. For the survival curves, statistical significance was determined using a log-rank (Mantel-Cox) test. Data for weights, systemic bacterial levels, antibiotic consumed and plasma enrofloxacin concentration were reported as mean ± SEM. A p<0.05 was considered significant.

## Results

### Systemic *Salmonella* levels are higher in VAD mice

**Fig 1:**
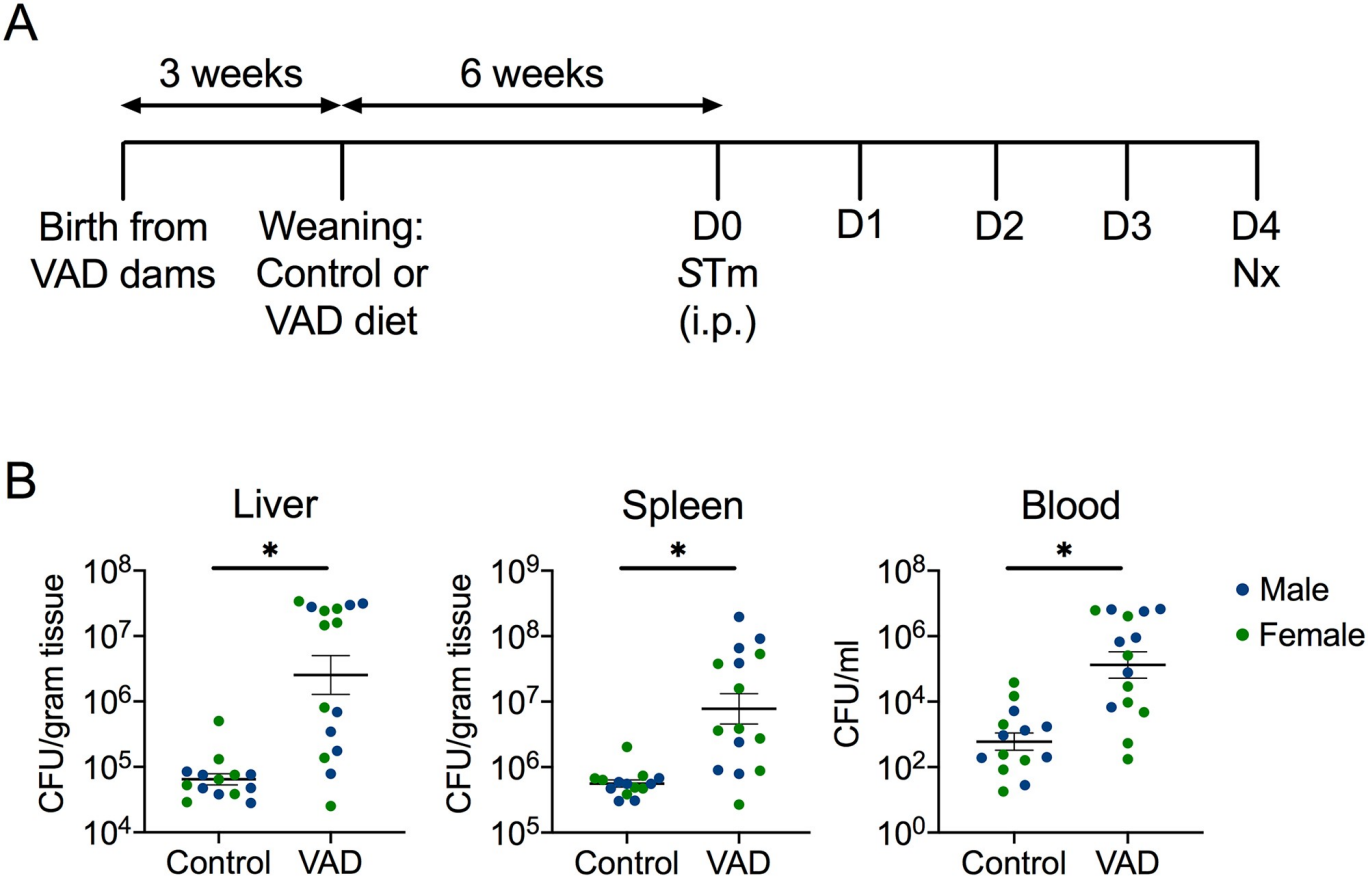
Systemic burden of *Salmonella* is higher in vitamin A-deficient mice. To generate vitamin A-deficient (VAD) mice, C57BL/6 *Slc11a1*^+/+^ pregnant mice were put on a VAD diet 2 weeks into gestation (**A**). At 3 weeks, pups were weaned onto either a VAD or control diet. Male (n = 7) and female (n = 7–8) mice on each diet were infected with *S*. Typhimurium (*S*Tm) D23580 via the intraperitoneal (i.p.) route at 9 weeks of age and systemic bacterial burden was characterized by colony-forming units (CFU) in liver, spleen and blood collected at necropsy (Nx) 4 days after infection (**B**). Data represent mean ± SEM. Significance between control and VAD mice was determined with a Mann-Whitney test of log-transformed values. A p<0.05 was considered significant.

In order to model how malnutrition and associated vitamin A deficiency are risk factors for developing systemic *S*. Typhimurium infection in people, we established a mouse model of vitamin A deficiency and disseminated disease (Fig 1A). Mice were maintained on either a VAD diet or a control diet containing adequate vitamin A. To model systemic infection, mice were infected with *S*. Typhimurium D23580 via the intraperitoneal (i.p.) route. At 4d post-infection, systemic levels of *S*. Typhimurium in liver, spleen and blood were significantly higher in VAD mice compared to control (Fig 1B), and there was no sex difference observed in bacterial colonization (S1A Fig). In a similar experiment with a necropsy at 5d post-infection, no sex differences in systemic bacterial burden was observed in control mice (S1B Fig). When combining data from mice necropsied at 4d and 5d post-infection, VAD mice of both sexes still had significantly higher systemic bacterial levels than control mice (S1C Fig). These data demonstrated that our model of vitamin A deficiency could be utilized to study its effect on susceptibility to systemic *S*. Typhimurium infection.

### Increased effect of VAD on *S*. Typhimurium-induced morbidity in male mice

**Fig 2:**
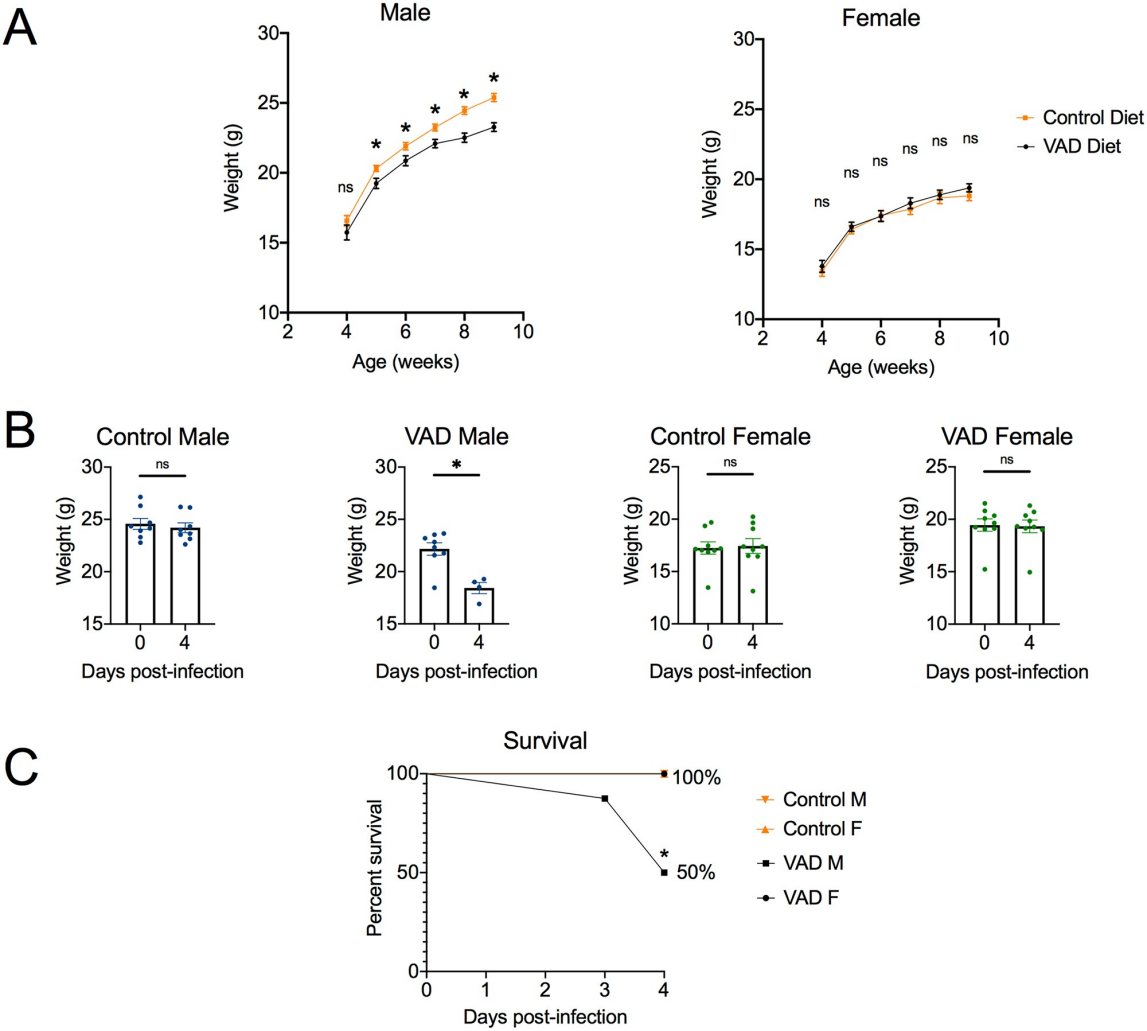
Increased weight loss and decreased survival seen in VAD male mice infected with *Salmonella*. Weight (g) of VAD and control male and female mice was recorded weekly (**A**) during growth from 4 weeks to 9 weeks (n = 26–30). Nine week-old mice were infected with *S*. Typhimurium D23580 via the intraperitoneal (i.p.) route and weight (g) was monitored daily (**B**) for up to 4 days post-infection (n = 8–9). Data are represented as mean ± SEM. Significance between weights by diet or day was determined using a Mann-Whitney test (*, p<0.05; ns, not significant). (**C**) Percent survival was assessed for all groups over the 4-day infection with a Kaplan-Meier survival curve (n = 8–9). Results are reported as percent survival. Pairwise comparisons between the VAD male group and each other group was determined with a survival analysis log-rank (Mantel-Cox) test (*, p<0.05). All data represent a compilation of data from three independent experiments.

It has been known for decades that vitamin A deficiency affects males and females differently [22]. To assess if this held true for our mouse model, both male and female mice were included. During maintenance on VAD and control diets, mice were weighed weekly. While diet had no effect on weight gain of female mice, VAD male mice gained significantly less weight than control mice starting at postnatal week 5 (Fig 2A). During the course of infection with *S*. Typhimurium D23580, VAD male mice had significant weight loss from day 0 to day 4 of infection, and 50% of mice in the VAD male group exhibited lethal morbidity by day 4 (Fig 2B and 2C). In contrast, VAD females and control mice of both sexes maintained their weight over the course of the experiment (Fig 2C).

While vitamin A deficiency had sex-specific effects on infection-induced morbidity (Fig 2B and 2C), both male and female mice were similar in exhibiting significantly higher systemic burdens of *S*. Typhimurium in VAD compared to control mice (S1C Fig). These results indicated that while vitamin A deficiency compromised control of systemic *S*. Typhimurium infection in both sexes, male mice were affected more strongly, both by reduced growth while on the VAD diet and by increased morbidity during infection.

### Sex-specific effects of vitamin A deficiency on responses to *S*. Typhimurium are apparent early after infection

**Fig 3:**
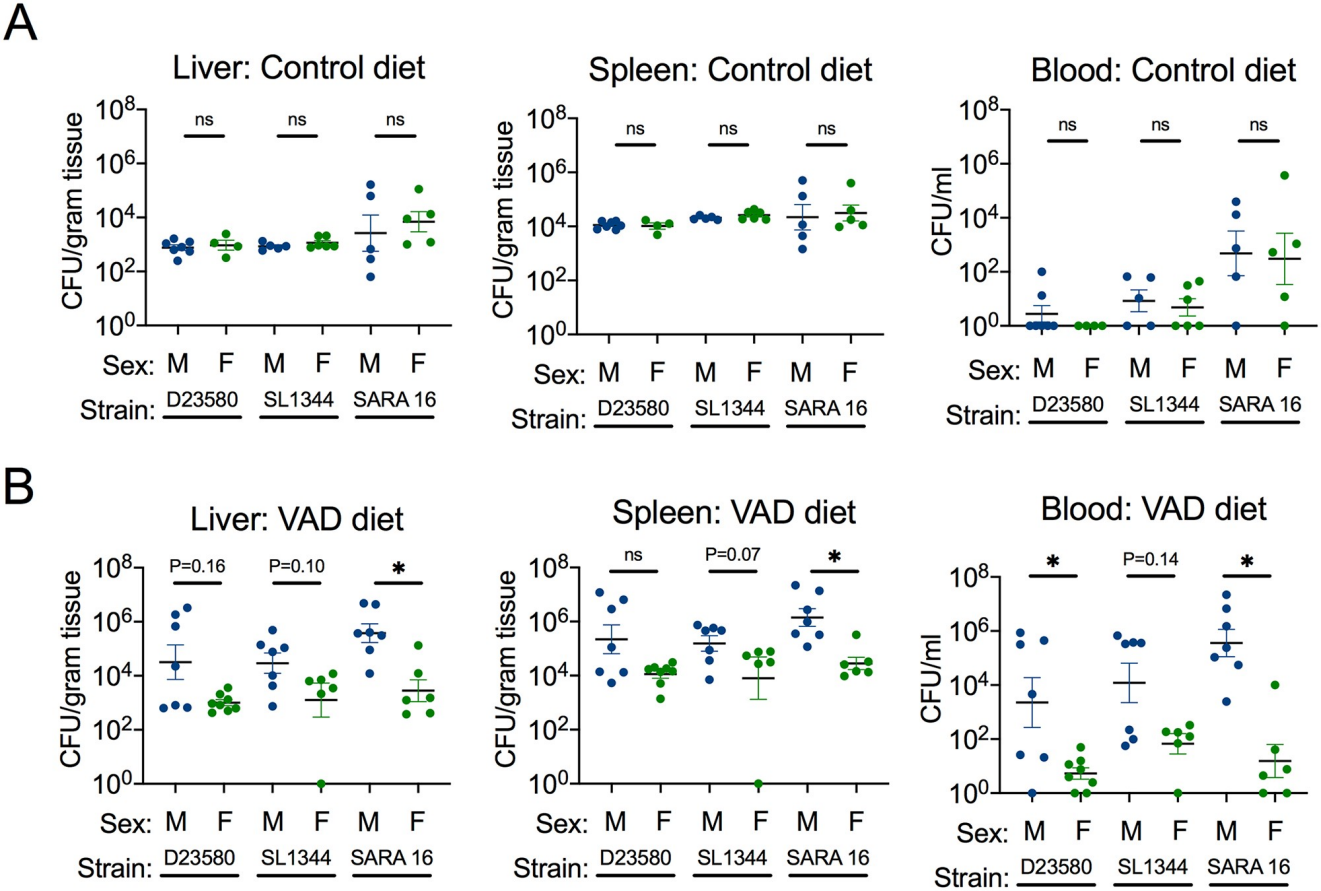
*Salmonella* strains colonize systemically at higher levels in VAD male mice early in infection. Systemic bacterial burden was characterized by CFU in liver, spleen and blood collected at necropsy 1 day after infection with *S*. Typhimurium D23580, SL1344 or SARA16 via the i.p. route for male and female mice (n = 4–8) on either control (**A**) or VAD (**B**) diet. Data represent mean ± SEM. Significance between male and female mice was determined with a Mann-Whitney test of log-transformed values (*, p<0.05; ns, not significant).

Since there was no sex difference in systemic bacterial burden by day 4 post-infection (Fig 1B) but VAD males lost weight and had decreased survival compared to female mice (Fig 2), we investigated whether an earlier time point would yield sex differences in *Salmonella* pathogenesis. Further, we assessed if this trend would hold true for multiple strains of *S*. Typhimurium, including D23580 (an ST313 strain), and the ST19 strains SL1344 and SARA16. There was no sex difference in systemic bacterial burden for any of the *S*. Typhimurium strains in mice fed a control diet (Fig 3A). However, at day 1 post-infection, male mice on a VAD diet had significantly more or a trend towards a higher systemic burden of all three *S*. Typhimurium strains than VAD female mice (Fig 3B). These results suggest that vitamin A deficiency has a more marked effect in male mice on responses that control systemic replication of *S*. Typhimurium in the early stages of infection.

### Antibiotic treatment failure occurs at specific concentrations *in vivo*

**Fig 4:**
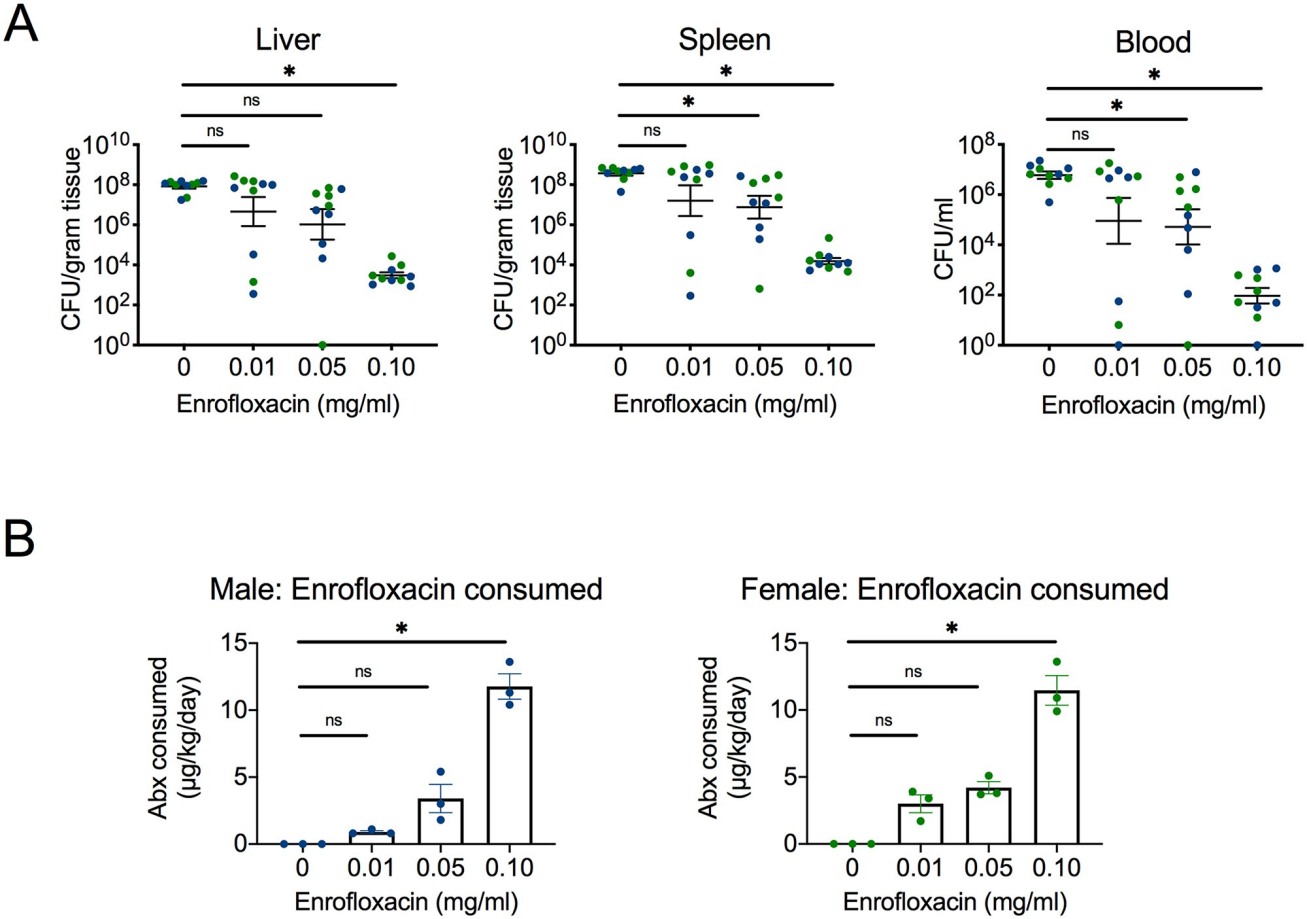
Antibiotic treatment failure occurs at specific concentrations *in vivo*. Enrofloxacin was administered directly into the drinking water on day 2 post-infection with multidrug-resistant *S*. Typhimurium D23580. Groups of C57BL/6J male (n = 5) and female (n = 5) mice on a standard diet were assessed for the following treatment groups: 0 mg/ml, 0.01 mg/ml, 0.05 mg/ml, and 0.10 mg/ml. Systemic bacterial burden was characterized by CFU in liver, spleen and blood collected at necropsy 5 days after infection (**A**). Water weights and mouse weights were collected daily. The average enrofloxacin consumed (μg/kg/day) was determined for each group based on water and mouse weights for days 2–3, 3–4 and 4–5 (**B**). Data are reported as mean ± SEM. Significance was determined on log-transformed values (**A**) or calculated values (**B**) with a Kruskal-Wallis test and Dunn’s multiple comparisons test (*, p<0.05; ns, not significant).

Emerging multidrug-resistant *S*. Typhimurium strains like D23580 are resistant to the first-line drugs for iNTS: ampicillin, chloramphenicol and trimethoprim/sulfamethoxazole. For this reason, the fluoroquinolone ciprofloxacin is one of the currently recommended treatments for iNTS [23]. However, emergence of fluoroquinolone resistance in *Salmonella* strains and host factors like malnutrition can contribute to treatment failure [24, 25]. We chose to model antibiotic treatment failure with the fluoroquinolone enrofloxacin since it can be administered orally through the drinking water to mice and because we could measure it in the bloodstream. We first confirmed susceptibility of D23580 to enrofloxacin *in vitro*. The average MIC was 0.04 μg/ml, which by CLSI guidelines is susceptible [18]. We next sought to determine an *in vivo* MIC. In order to assess the subtherapeutic concentration of enrofloxacin administered in the drinking water, systemic bacterial levels were assessed for male and female mice at a range of concentrations at day 5 post-infection. To model antibiotic treatment failure, enrofloxacin administration was delayed to day 2 post-infection. We used C57BL/6J mice fed a standard diet for titration experiments. No sex difference was observed in systemic bacterial levels (S2 Fig). A significant decrease in bacteria in liver, spleen and blood was seen at an enrofloxacin concentration of 0.10 mg/ml (Fig 4A). The concentration 0.01 mg/ml was subtherapeutic, as it failed to significantly decrease systemic *S*. Typhimurium levels. The concentration 0.05 mg/ml was partly subtherapeutic, as it did not significantly decrease *S*. Typhimurium levels in the liver. Water weights and mouse weights were monitored daily during infection, and the average amount of enrofloxacin consumed was calculated in μg/kg/day for days 2–3, 3–4 and 4–5. Enrofloxacin consumption tracked with dosage, although 0.01 mg/ml and 0.05 mg/ml were not statistically significant from the untreated group (Fig 4B). Taken together, these data demonstrated that 0.01 mg/ml and 0.05 mg/ml could model antibiotic treatment failure and thus were used for subsequent experiments with mice on special diets.

### Vitamin A and subtherapeutic antibiotic co-treatment synergize to decrease systemic *S*. Typhimurium levels and improve survival in VAD male mice

**Fig 5:**
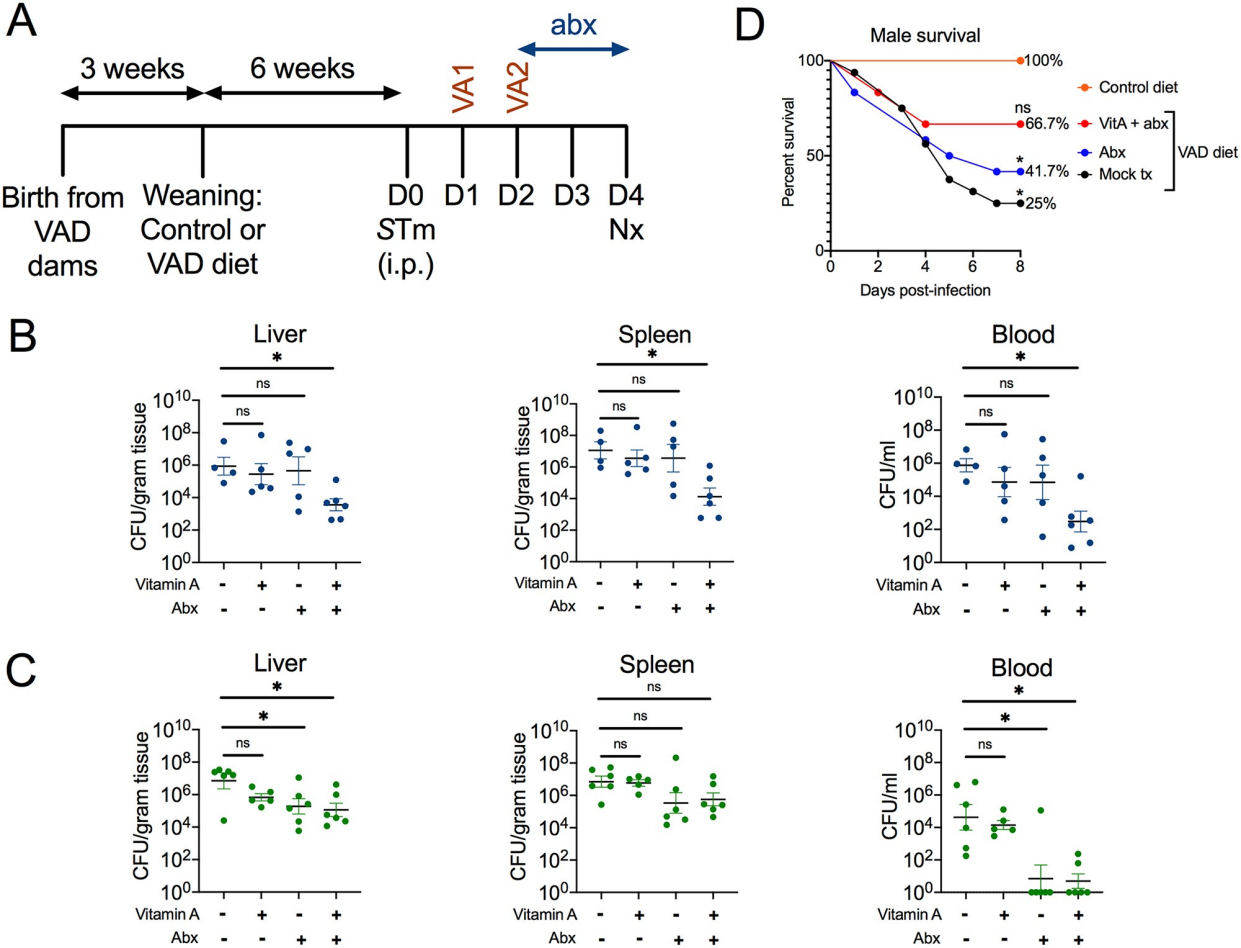
Co-treatment of vitamin A and antibiotics decreases systemic *Salmonella* burden and improves survival in VAD male mice. VAD and control *Slc11a1*^*+/+*^ mice were generated and infected with multidrug resistant *S*. Typhimurium D23580 at 9 weeks of age. Mock-treated mice received 100 μl of sterile PBS administered by oral gavage on day 1 and day 2 post-infection. Vitamin A-treated mice received 600 IU of retinyl palmitate in 100 μl of sterile PBS administered by oral gavage on day 1 and day 2 post-infection and changed to control diet at day 1 post-infection. The antibiotic enrofloxacin (abx) was administered at day 2 post-infection in the drinking water at 0.05 mg/ml (**A**). Systemic burden of *S*. Typhimurium was characterized for VAD male (**B**) and VAD female (**C**) mice (n = 4–6) as CFU in liver, spleen and blood at necropsy 4 days post-infection. Data are reported as mean ± SEM. Significance was determined on log-transformed values with a Kruskal-Wallis test and Dunn’s multiple comparisons test (*, p<0.05; ns, not significant). (**D**) Percent survival for control and treated VAD male mice was assessed with an 8-day Kaplan-Meier survival curve (n = 6–16). An enrofloxacin concentration of 0.01 mg/ml was used in drinking water treatment, and oral gavage treatments were given as previously described. Significance was determined with pairwise comparisons between the control group and each other treatment group using a log-rank (Mantel-Cox) test (*, p<0.05; ns, not significant). Data from untreated controls are also included in Fig 1.

To assess the therapeutic potential of vitamin A in the context of antibiotic treatment failure, systemic levels of *S*. Typhimurium D23580 at day 4 post-infection were assessed for male and female mice on either control or VAD diets with the following treatment groups: mock-treated, vitamin A only, enrofloxacin (0.05 mg/ml) only, and vitamin A and enrofloxacin co-treatment (Fig 5A). In VAD male mice, co-treatment significantly decreased bacterial levels at all three systemic sites in comparison to mock treatment; however, individual treatments did not (Fig 5B). Systemic colonization of VAD male mice in all groups combined was significantly higher if the mouse had >10% weight loss than if the mouse had <10% weight loss (S3 Fig). Mock-treated VAD male mice lost significant weight from day 0 to day 4, but none of the treatment groups were significant (S4 Fig). In contrast, VAD female mice either showed no significant improvement with any treatment (spleen), or equal effectiveness with enrofloxacin alone and co-treatment (liver, blood) (Fig 5C). In male and female mice on a control diet, both enrofloxacin (0.05 mg/ml) and co-treatment yielded significant or trending decreases in systemic *S*. Typhimurium (S5A and S5B Fig). Since 0.05 mg/ml enrofloxacin was successful, independent of vitamin A, at decreasing *S*. Typhimurium in liver and blood in VAD female mice and control mice for both sexes, a concentration of 0.01 mg/ml was also assessed for these groups. However, co-treatment of 0.01 mg/ml enrofloxacin and vitamin A yielded no benefit in VAD female mice compared to mock-treated animals (S6 Fig). For males on a control diet, co-treatment of 0.01 mg/ml enrofloxacin and vitamin A showed a slight decrease in splenic bacterial levels, but there was no difference in the liver and blood (S7A Fig). There was no benefit of any therapy for females on a control diet (S7B Fig).

Enrofloxacin is a common antibiotic used in veterinary medicine, and it is converted to ciprofloxacin by the liver [26]. No significant differences in plasma enrofloxacin or plasma ciprofloxacin levels were detected between enrofloxacin only and co-treated male (S8A Fig) and female (S8B Fig) mice, as assessed by LC-MS/MS. These results ruled out the possibility that vitamin A could work by increasing consumption or bioavailability of the antibiotic.

As 0.01 mg/ml was subtherapeutic such that there was still lethal morbidity in VAD male mice, these conditions were used to assess survival of VAD male mice with treatments. Mice were monitored for 8 days post-infection and euthanized upon showing signs of lethal morbidity, and a Kaplan-Meier survival curve was generated (Fig 5D). Of the infected mice fed control diets, 100% of males survived. The percent survival of mock-treated VAD males was only 25%, but this was improved with enrofloxacin only (41.7%) or co-treatment of vitamin A and enrofloxacin (66.7%). Significance between each treatment group and survival of mice on a control diet was assessed with a log-rank (Mantel-Cox) test. Further, for the 44 male mice represented in the survival curve (Fig 5D), weight loss correlated with survival. Over the course of the experiment, if a mouse lost less than 10% of its weight, it was more likely to have survived (16/21; 76% survival) than if a mouse had lost more than 10% of its weight (7/23; 30% survival). In a separate experiment with a longer time course grouping sexes, vitamin A treatment alone improved survival by 66.7% in VAD mice (S9 Fig). Taken together, these data indicated that vitamin A synergized with subtherapeutic (0.05 mg/ml) enrofloxacin treatment to decrease systemic *S*. Typhimurium levels and improve survival in VAD male mice.

## Discussion

Epidemiologic studies have shown that vitamin A deficiency and disseminated non-typhoidal *Salmonella* infections are co-endemic, disproportionately affecting sub-Saharan Africa [10, 27]. The high mortality rate of disseminated infection has been attributed to antimicrobial resistance of ST313 strains that circulate regionally in sub-Saharan Africa, to genomic features of these strains associated with increased systemic dissemination, and to the high prevalence of co-morbidities that predispose to systemic disease [17, 23, 24]. The current study was designed as a preclinical trial in mice to assess the potential utility of vitamin A as an adjunct to antibiotic therapy. Our results suggest a benefit of combined antibiotic treatment and vitamin A supplementation, with the effect being most apparent in male mice.

It is well-appreciated that vitamin A supplementation can be a useful tool for disease prevention and treatment. In populations with a ≥1% prevalence of night blindness or a ≥20% prevalence of vitamin A deficiency, the WHO recommends vitamin A supplementation for children 12–59 months of age [28]. Vitamin A supplementation was found to significantly reduce the risk of all-cause morbidity and mortality due to diarrhea [29]. In children 2 and under presenting with measles, the WHO-recommended treatment of two consecutive daily megadoses of vitamin A [30] reduces overall and pneumonia-specific mortality [11] and decreases duration of diarrhea and fever. However, the utility of vitamin A for bacterial bloodstream infections has not been assessed. *S*. Typhimurium bloodstream infections typically present as a febrile illness; diarrhea is often absent or not a prominent symptom [10, 31, 32]. Our results suggest that co-treatment with vitamin A and antibiotics may improve outcomes during bloodstream infections with drug-resistant *S*. Typhimurium. Despite WHO’s recommendation for vitamin A supplementation, full coverage is still lacking in many countries. Therefore, supplementing with vitamin A during episodes of febrile illness is a potential strategy to increase coverage and improve infection outcomes.

A notable finding of our study was the sex-specific effect of vitamin A supplementation with antibiotics for treatment of *S*. Typhimurium infection in mice. The picture emerging from recent studies of innate immunity is that multiple sex-specific differences impact resistance of males and females to infection. Human males are more susceptible to many infectious diseases, whereas females are more at risk of developing an autoimmune condition [33]. In current events, males are at a higher risk of severe COVID-19 infection [34]. Males also have a higher risk of developing sepsis [35, 36]. A study on sepsis in children found that vitamin A deficiency was more common in the sepsis group as compared to control, and vitamin A deficiency rates were ~80% in the subgroups of severe sepsis and septic shock [37], indicating how a VAD status can predispose to severe disease. The majority of patients with sepsis in this study were male. In addition, some genes encoding for innate immune molecules are located on the X chromosome [33], suggesting a genetic basis for some of the differences observed. It is therefore possible that malnutrition and vitamin A deficiency may compromise the immune response to systemic *Salmonella* infection in a sex-specific manner. We speculate that vitamin A may be playing the role of a host-directed therapy by boosting the immune response of VAD male mice, synergizing with an initially subtherapeutic dose of antibiotic, and leading to better immunological control of infection. While the current study did not investigate the mechanisms underlying sex differences in the response to vitamin A supplementation, possible explanations include sex differences in innate immune cell function [33], intestinal microbiota composition [38], and hormone levels [39].

Antibiotic treatment failure is the persistence of symptoms despite initiation of antibiotics. Common causes include wrong diagnosis, infection with a resistant organism, or host failure such as malnutrition [25]. We modeled antibiotic treatment failure with a subtherapeutic enrofloxacin concentration that yielded no difference in systemic bacterial burden, but circulating antibiotic was still present in the mouse. It can be the joint action of antibiotics and host defense mechanisms that leads to clearance of bacteria and resolution of disease. While antibiotics are typically studied for their bactericidal or bacteriostatic activity against susceptible bacteria, they can also exist at subtherapeutic or subinhibitory concentrations, i.e. at concentrations below the MIC. Subinhibitory antibiotic concentrations can still affect both the host and the bacterium [40]. It has been proposed that some antibiotics may influence antiphagocytic cell-surface structures and lead to enhanced phagocytosis, modifying immune cell function [41]. For example, neutrophil phagocytosis and intracellular killing activity have been enhanced by pre-incubation with subinhibitory antimicrobial concentrations in some studies, although it depends on the antibiotic used and organism studied [42]. One study reported that subinhibitory levels of fluoroquinolone antibiotics like ciprofloxacin lead to enhanced binding of anti-LPS antibodies to Enterobacteriaceae like *Escherichia coli* [43]. Taken together, these findings suggest that vitamin A treatment may synergize with subtherapeutic antibiotics to enhance immune function in male mice.

Our study has limitations. We studied the effect of vitamin A co-treatment with only a single antibiotic, enrofloxacin, and a single clinical isolate, D23580. Common treatments for iNTS have included first-line antibiotics of ampicillin, chloramphenicol and trimethoprim/sulfamethoxazole [23]. D23580 is resistant to all of these first-line drugs. It is sensitive to fluoroquinolones at certain concentrations *in vitro* and *in vivo*. However, future studies should incorporate *S*. Typhimurium strains also resistant to fluoroquinolones, as this is an emerging issue [9]. In addition, it would be important to determine whether vitamin A supplementation can prevent treatment failure with other commonly used antibiotics. There are also limitations to using a mouse model of infection, including the low genetic variation of inbred mice used and the short infection time course permitted by the VAD male mouse model. Future studies could incorporate additional mouse lines, routes of infection and challenge doses. However, given the track record of using vitamin A as prevention and treatment for other infections, this information on whether vitamin A supplementation could be effective against disseminated infections with antibiotic-resistant *Salmonella* might be best obtained in a clinical study.

## Supporting information

S1 Fig Systemic burden of *Salmonella* in male and female mice on control and VAD diets. **A**. Data in Fig 1B were re-analyzed to compare *S*. Typhimurium colonization level in each group by sex (n = 7–8). **B**. Levels of *S*. Typhimurium in liver, spleen and blood at day 5 post-infection in male and female mice (n = 1–7). The same data is represented in two different ways. Initially there were more VAD male mice in the study but they reached lethal endpoint prior to day 5 and were likely to have a higher CFU than in the figure. **C**. Levels of *S*. Typhimurium in liver, spleen and blood at day 4 and 5 post-infection combined (n = 8–15). Data shown in this figure are a re-analysis of data presented in **S1A** and **S1B**. Data represent mean ± SEM. Significance between groups was determined with a Mann-Whitney test of log-transformed values (*, p<0.05; ns, not significant).
(TIF)

S2 Fig Systemic burden of *Salmonella* in male and female mice during antibiotic treatment. Relating to Fig 4A, comparison of levels of *S*. Typhimurium in liver, spleen and blood at day at day 5 post-infection in male and female mice on a standard diet after antibiotic treatment (n = 5). Data represent mean ± SEM. Significance between male and female mice was determined with a Mann-Whitney test of log-transformed values (*, p<0.05; ns, not significant).
(TIF)

S3 Fig Systemic burden of *Salmonella* as compared to percent weight lost by VAD male mice during infection. Relating to VAD male mice in Fig 5B, systemic colonization of mice in all treatment groups combined grouped by <10% weight loss (n = 14) versus >10% weight loss (n = 12) by day 4 post-infection with *S*. Typhimurium D23580. Data represent mean ± SEM. Significance between weight loss groups was determined with a Mann-Whitney test of log-transformed values. A p<0.05 was considered significant.
(TIF)

S4 Fig Weight change of mock-treated and treated VAD male mice during infection with *Salmonella*. Relating to VAD male mice (n = 4–6) in Fig 5B, weight change during infection with *S*. Typhimurium D23580 during mock treatment, treatment with vitamin A only, treatment with 0.05 mg/ml enrofloxacin in the drinking water only, or vitamin A and antibiotic co-treatment. Data represent mean ± SEM. Significance between day 0 and day 4 weights was determined with a Mann-Whitney test (*, p<0.05; ns, not significant).
(TIF)

S5 Fig Systemic burden of *Salmonella* in male and female mice on a control diet during treatment with 0.05 mg/ml enrofloxacin. ***A***. Levels of *S*. Typhimurium in liver, spleen and blood at day 4 post-infection in male mice (n = 3–6) on a control diet administered either mock treatment, vitamin A only, 0.05 mg/ml enrofloxacin only, or co-treatment. ***B***. Levels of *S*. Typhimurium in liver, spleen and blood at day 4 post-infection in female mice (n = 4–7) on a control diet administered either mock treatment, vitamin A only, 0.05 mg/ml enrofloxacin only, or co-treatment. Data represent mean ± SEM. Significance was determined on log-transformed values with a Kruskal-Wallis test and Dunn’s multiple comparisons test (*, p<0.05; ns, not significant). Data from untreated control mice are also shown in Fig 1.
(TIF)

S6 Fig Systemic burden of *Salmonella* in female mice on a VAD diet during treatment with 0.01 mg/ml enrofloxacin. Levels of *S*. Typhimurium in liver, spleen and blood at day 4 post-infection in female mice (n = 4–6) on a VAD diet administered either mock treatment, vitamin A only, 0.01 mg/ml enrofloxacin only, or co-treatment. VAD males treated with 0.01 mg/ml showed decreased survival and are not shown. Data represent mean ± SEM. Significance was determined on log-transformed values with a Kruskal-Wallis test and Dunn’s multiple comparisons test (*, p<0.05; ns, not significant).
(TIF)

S7 Fig Systemic burden of *Salmonella* in male and female mice on a control diet during treatment with 0.01 mg/ml enrofloxacin. **A**. Levels of *S*. Typhimurium in liver, spleen and blood at day 4 post-infection in male mice (n = 3–4) on a control diet administered either mock treatment, vitamin A only, 0.01 mg/ml enrofloxacin only, or co-treatment. **B**. Levels of *S*. Typhimurium in liver, spleen and blood at day 4 post-infection in female mice (n = 4–7) on a control diet administered either mock treatment, vitamin A only, 0.01 mg/ml enrofloxacin only, or co-treatment. Data represent mean ± SEM. Significance was determined on log-transformed values with a Kruskal-Wallis test and Dunn’s multiple comparisons test (*, p<0.05; ns, not significant).
(TIF)

S8 Fig Concentrations of antibiotic in the plasma during treatment with 0.05 mg/ml enrofloxacin. ***A***. Levels of enrofloxacin and ciprofloxacin (ng/ml) in the plasma during mock treatment, 0.05 mg/ml enrofloxacin in the drinking water, or co-treatment of 0.05 mg/ml enrofloxacin and vitamin A in male mice (n = 1–2) on control or VAD diet, as assessed by LC-MS/MS. ***B***. Levels of enrofloxacin and ciprofloxacin (ng/ml) in the plasma during mock treatment, 0.05 mg/ml enrofloxacin in the drinking water, or co-treatment of 0.05 mg/ml enrofloxacin and vitamin A in female mice (n = 3–4) on control or VAD diet, as assessed by LC-MS/MS. Data represent mean ± SEM. Significance between antibiotic levels in antibiotic-treated and co-treated mice was determined with a Mann-Whitney test (*, p<0.05; ns, not significant).
(TIF)

S9 Fig Survival of mice when treated with vitamin A only over a 13-day time course. Survival of grouped male and female mice treated with two consecutive doses of either PBS or retinyl palmitate (600 IU delivered by oral gavage) starting 1 day after *S*. Typhimurium infection. Additionally, VAD mice treated with retinyl palmitate were placed on a control diet replete with vitamin A one-day after *S*. Typhimurium infection. Data represent percent survival of 12 mice per group from three independent experiments. Pairwise comparisons between the control diet group and each other group was determined with a survival analysis log-rank (Mantel-Cox) test (*, p<0.05).
(TIF)
