# Supplementary figures and images for: Vitamin A supplementation boosts control of antibiotic-resistant Salmonella infection in malnourished mice
Source: PLoS Negl Trop Dis. 2020 Oct 2;14(10):e0008737. doi: 10.1371/journal.pntd.0008737 (PMC7556496; doi:10.1371/journal.pntd.0008737)

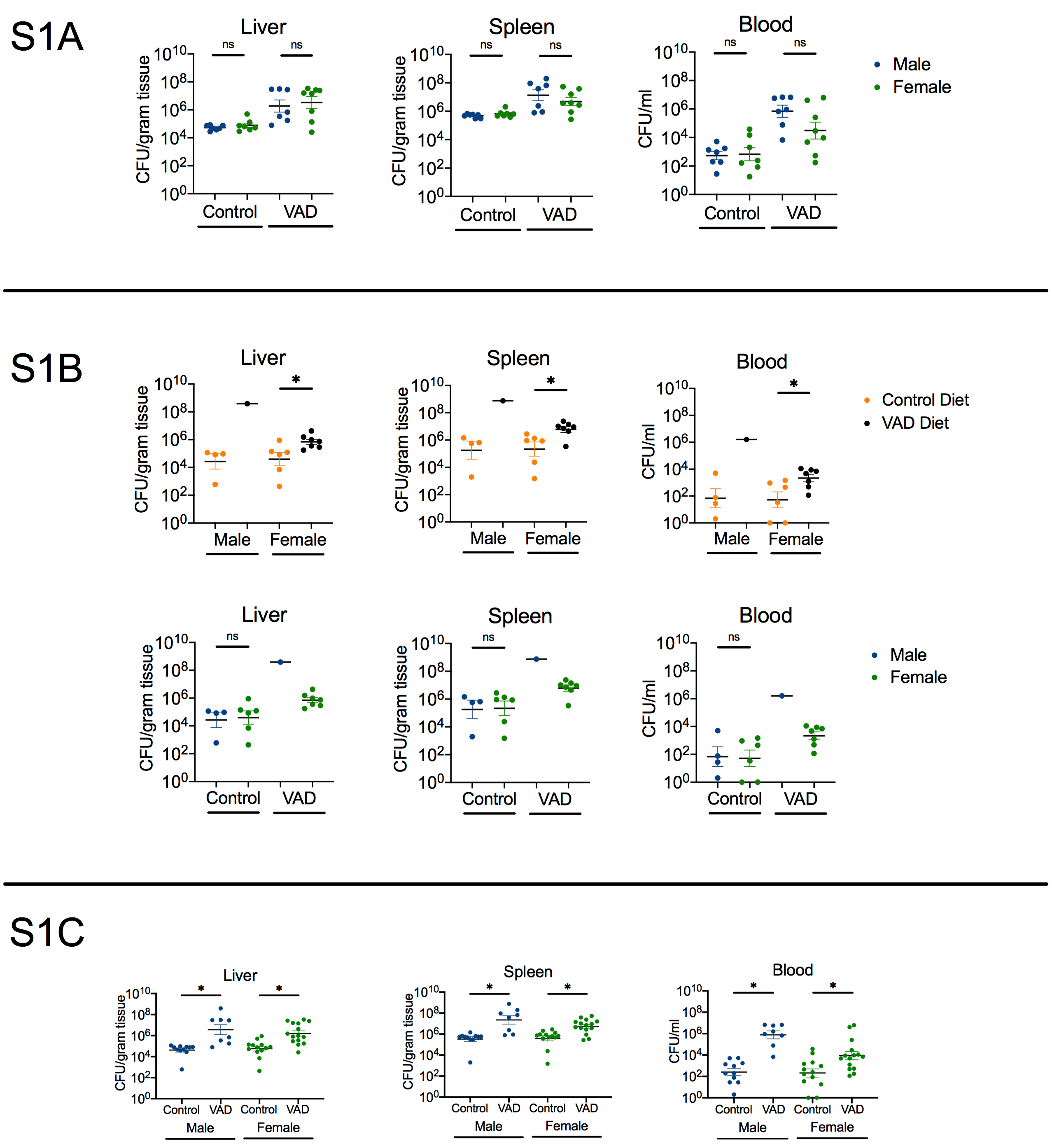

Supplement: S1 Fig — A. Data in Fig 1B were re-analyzed to compare S. Typhimurium colonization level in each group by sex (n = 7–8). B. Levels of S. Typhimurium in liver, spleen and blood at day 5 post-infection in male and female mice (n = 1–7). The same data is represented in two different ways. Initially there were more VAD male mice in the study but they reached lethal endpoint prior to day 5 and were likely to have a higher CFU than in the figure. C. Levels of S. Typhimurium in liver, spleen and blood at day 4 and 5 post-infection combined (n = 8–15). Data shown in this figure are a re-analysis of data presented in S1A and S1B. Data represent mean ± SEM. Significance between groups was determined with a Mann-Whitney test of log-transformed values (*, p<0.05; ns, not significant). (TIF) [file pntd.0008737.s001.tif]

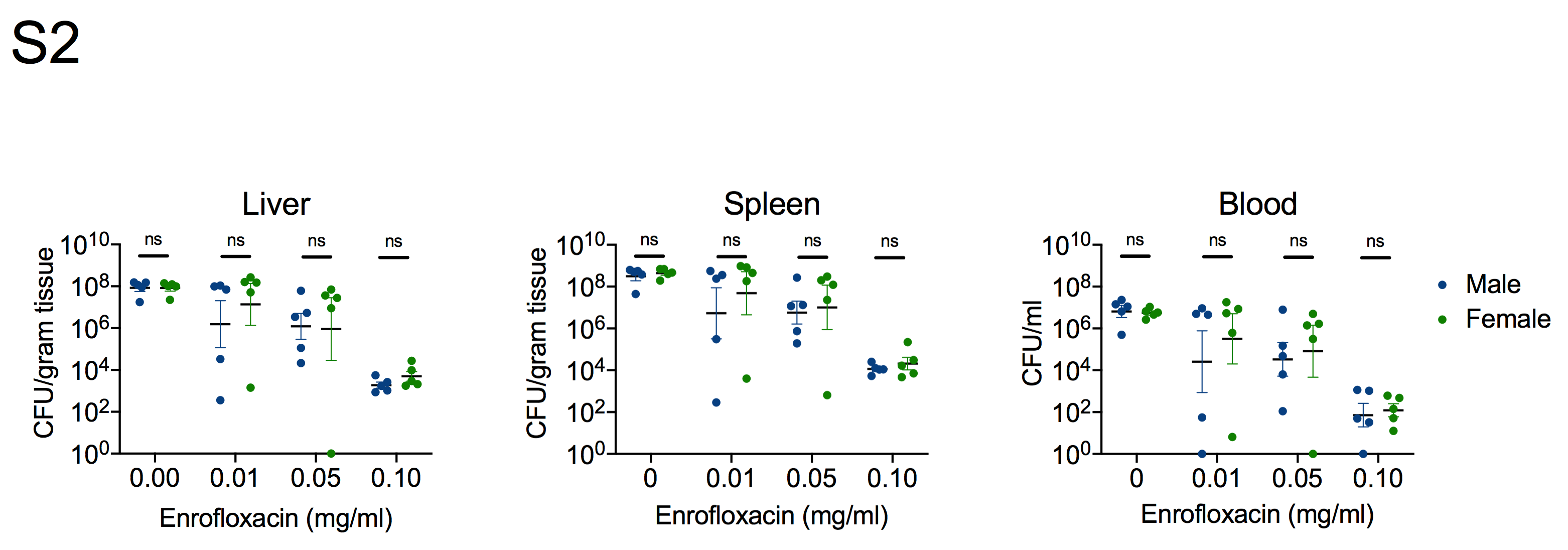

Supplement: S2 Fig — Relating to Fig 4A, comparison of levels of S. Typhimurium in liver, spleen and blood at day at day 5 post-infection in male and female mice on a standard diet after antibiotic treatment (n = 5). Data represent mean ± SEM. Significance between male and female mice was determined with a Mann-Whitney test of log-transformed values (*, p<0.05; ns, not significant). (TIF) [file pntd.0008737.s002.tif]

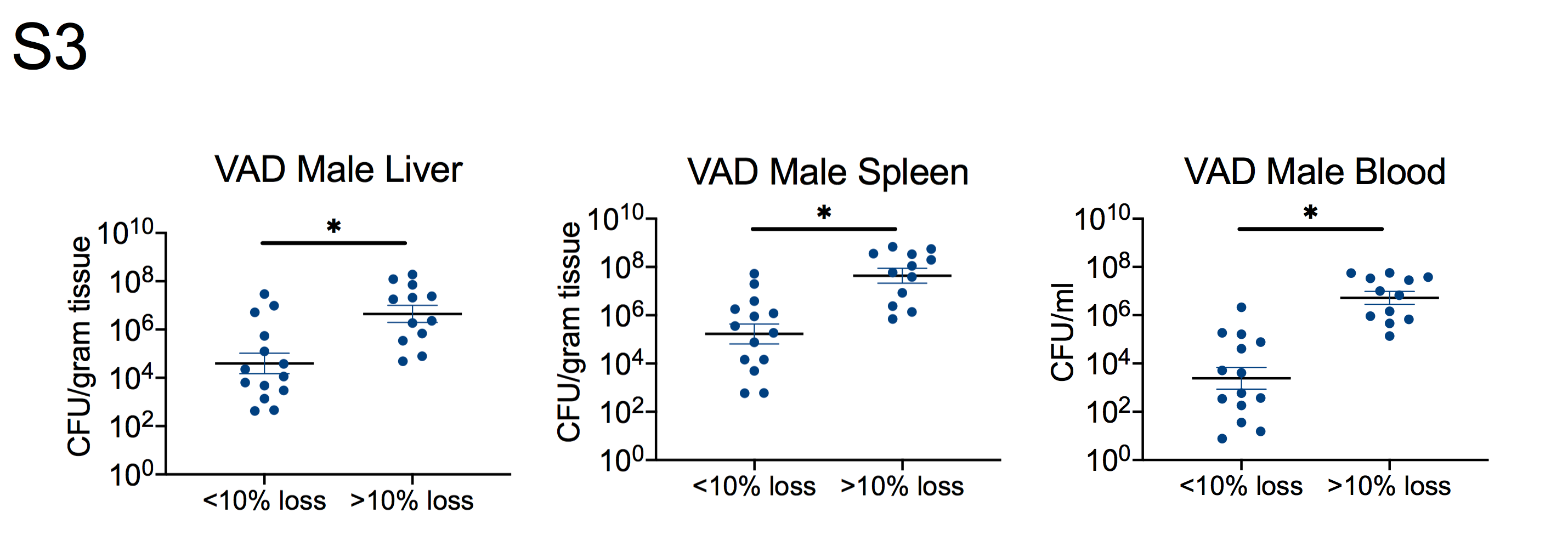

Supplement: S3 Fig — Relating to VAD male mice in Fig 5B, systemic colonization of mice in all treatment groups combined grouped by <10% weight loss (n = 14) versus >10% weight loss (n = 12) by day 4 post-infection with S. Typhimurium D23580. Data represent mean ± SEM. Significance between weight loss groups was determined with a Mann-Whitney test of log-transformed values. A p<0.05 was considered significant. (TIF) [file pntd.0008737.s003.tif]

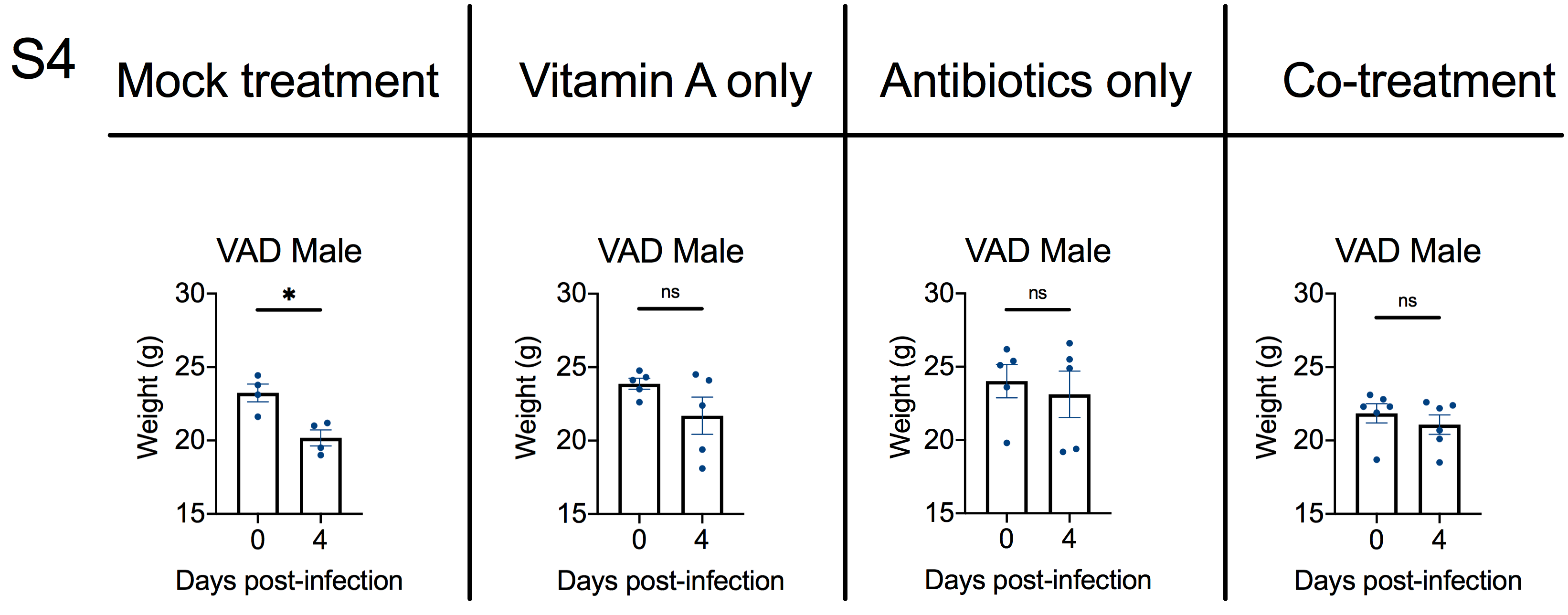

Supplement: S4 Fig — Relating to VAD male mice (n = 4–6) in Fig 5B, weight change during infection with S. Typhimurium D23580 during mock treatment, treatment with vitamin A only, treatment with 0.05 mg/ml enrofloxacin in the drinking water only, or vitamin A and antibiotic co-treatment. Data represent mean ± SEM. Significance between day 0 and day 4 weights was determined with a Mann-Whitney test (*, p<0.05; ns, not significant). (TIF) [file pntd.0008737.s004.tif]

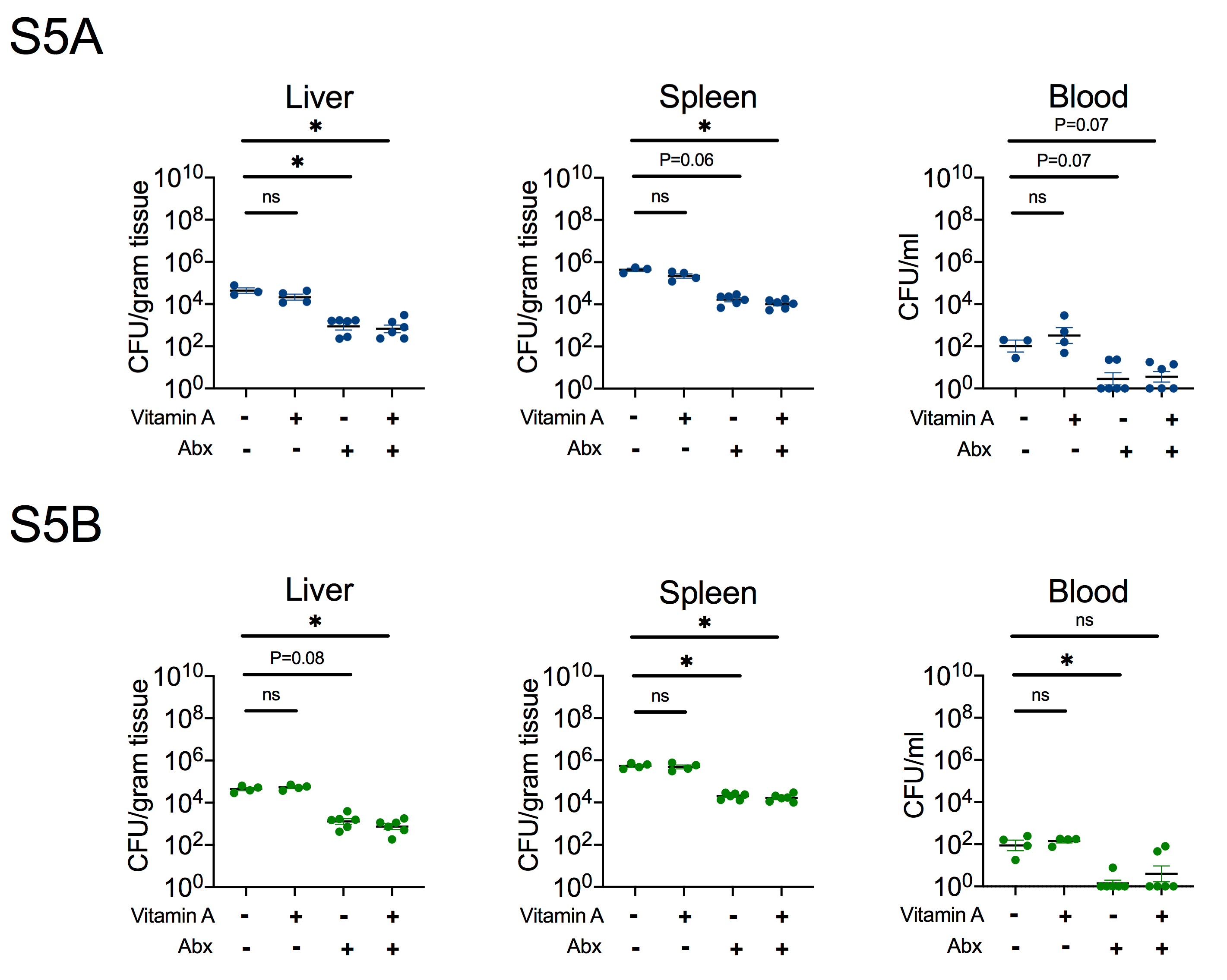

Supplement: S5 Fig — A. Levels of S. Typhimurium in liver, spleen and blood at day 4 post-infection in male mice (n = 3–6) on a control diet administered either mock treatment, vitamin A only, 0.05 mg/ml enrofloxacin only, or co-treatment. B. Levels of S. Typhimurium in liver, spleen and blood at day 4 post-infection in female mice (n = 4–7) on a control diet administered either mock treatment, vitamin A only, 0.05 mg/ml enrofloxacin only, or co-treatment. Data represent mean ± SEM. Significance was determined on log-transformed values with a Kruskal-Wallis test and Dunn’s multiple comparisons test (*, p<0.05; ns, not significant). Data from untreated control mice are also shown in Fig 1. (TIF) [file pntd.0008737.s005.tif]

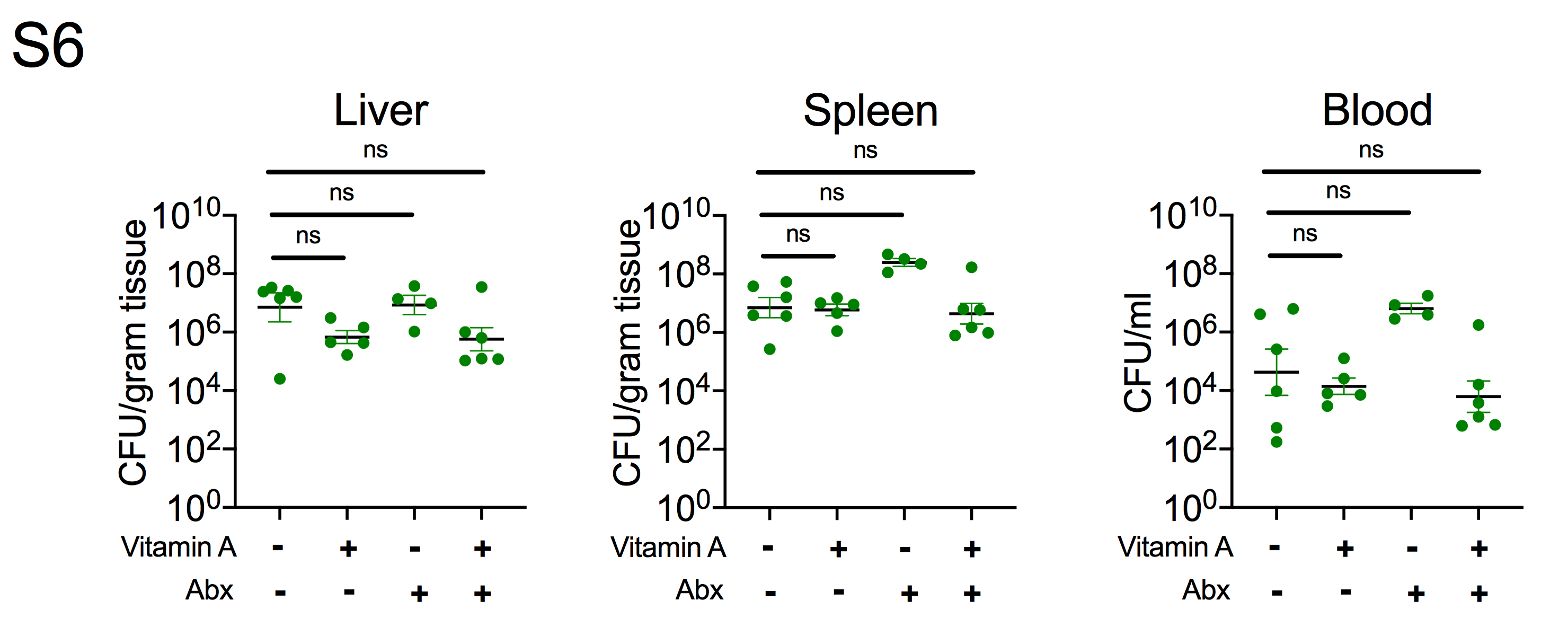

Supplement: S6 Fig — Levels of S. Typhimurium in liver, spleen and blood at day 4 post-infection in female mice (n = 4–6) on a VAD diet administered either mock treatment, vitamin A only, 0.01 mg/ml enrofloxacin only, or co-treatment. VAD males treated with 0.01 mg/ml showed decreased survival and are not shown. Data represent mean ± SEM. Significance was determined on log-transformed values with a Kruskal-Wallis test and Dunn’s multiple comparisons test (*, p<0.05; ns, not significant). (TIF) [file pntd.0008737.s006.tif]

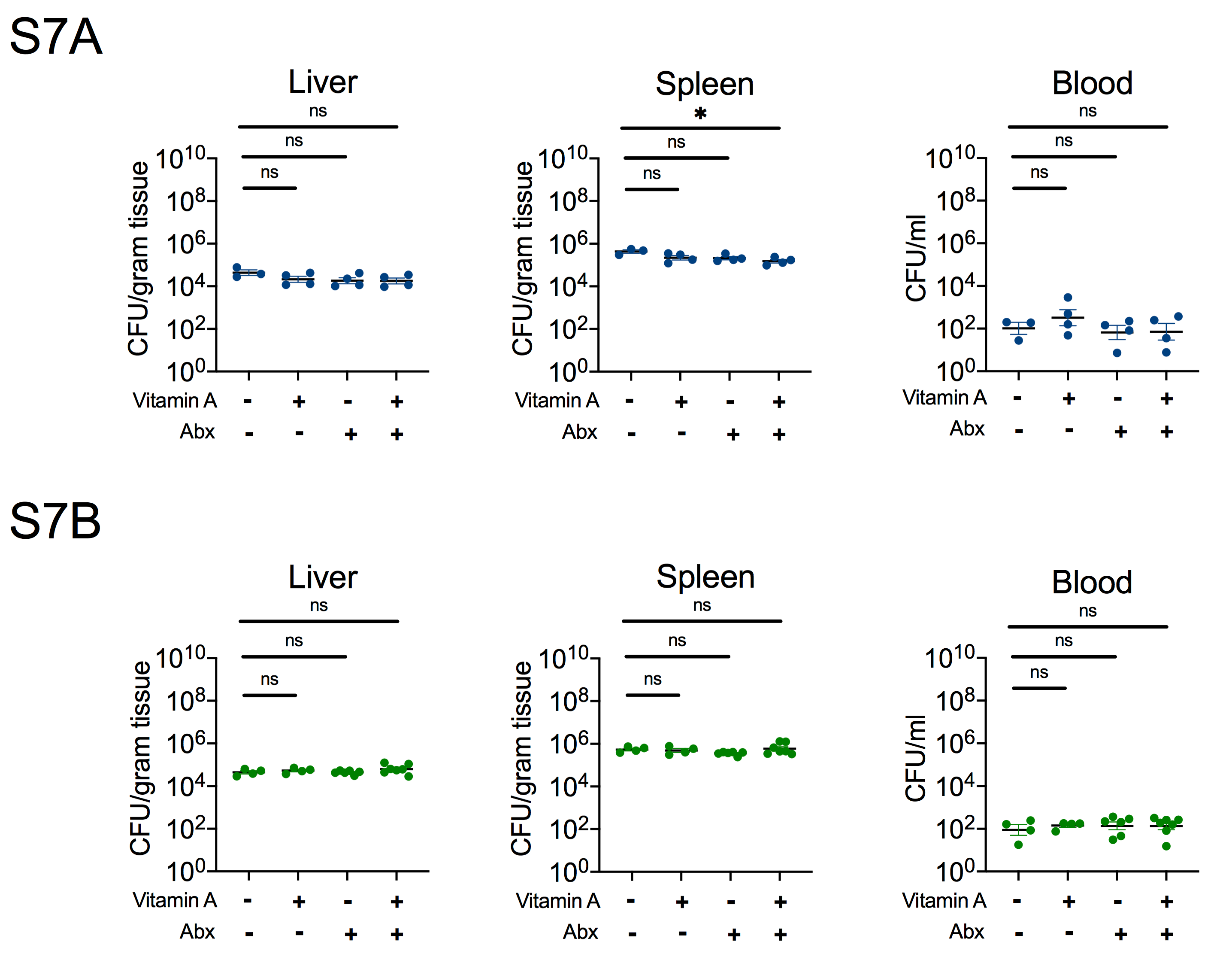

Supplement: S7 Fig — A. Levels of S. Typhimurium in liver, spleen and blood at day 4 post-infection in male mice (n = 3–4) on a control diet administered either mock treatment, vitamin A only, 0.01 mg/ml enrofloxacin only, or co-treatment. B. Levels of S. Typhimurium in liver, spleen and blood at day 4 post-infection in female mice (n = 4–7) on a control diet administered either mock treatment, vitamin A only, 0.01 mg/ml enrofloxacin only, or co-treatment. Data represent mean ± SEM. Significance was determined on log-transformed values with a Kruskal-Wallis test and Dunn’s multiple comparisons test (*, p<0.05; ns, not significant). (TIF) [file pntd.0008737.s007.tif]

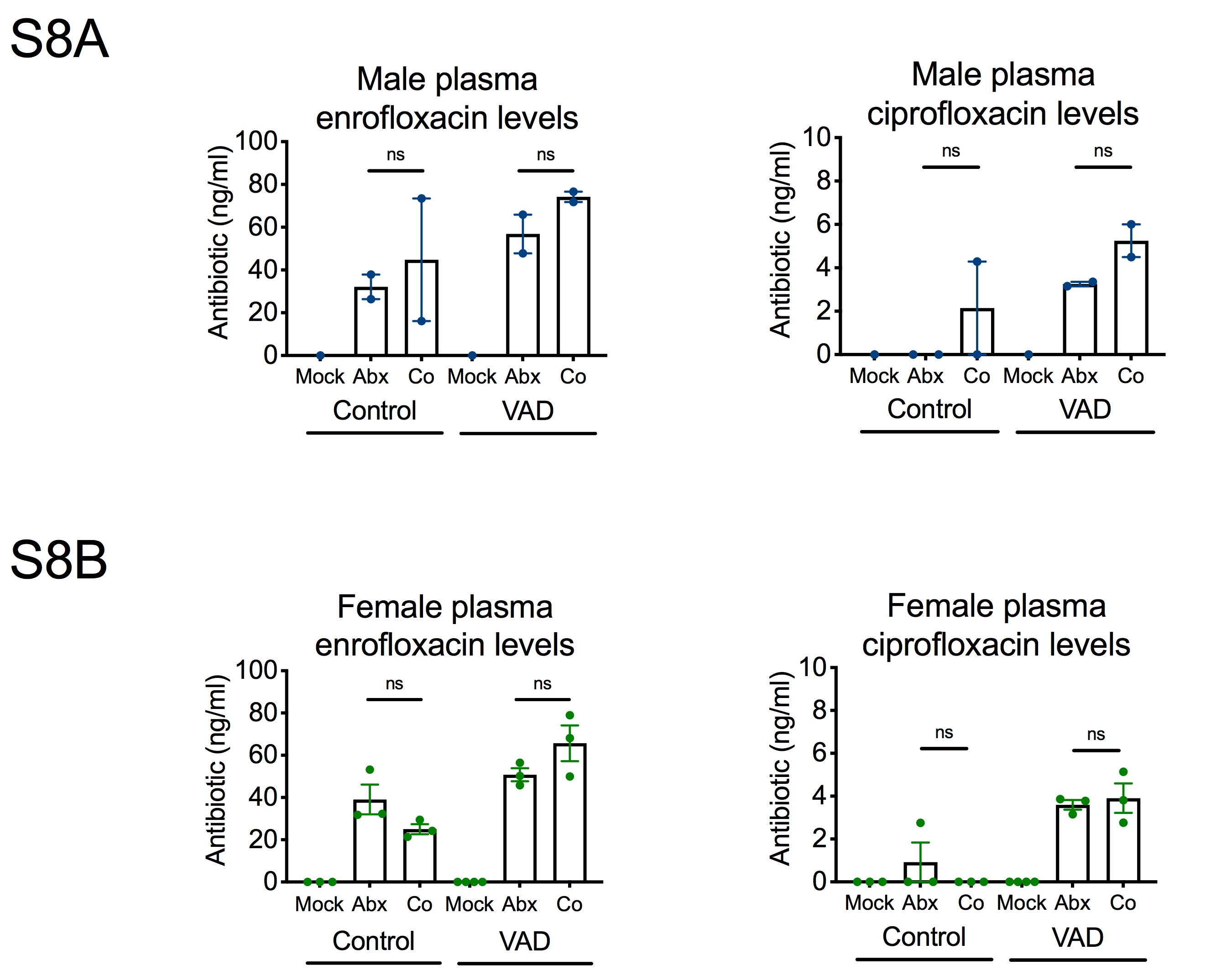

Supplement: S8 Fig — A. Levels of enrofloxacin and ciprofloxacin (ng/ml) in the plasma during mock treatment, 0.05 mg/ml enrofloxacin in the drinking water, or co-treatment of 0.05 mg/ml enrofloxacin and vitamin A in male mice (n = 1–2) on control or VAD diet, as assessed by LC-MS/MS. B. Levels of enrofloxacin and ciprofloxacin (ng/ml) in the plasma during mock treatment, 0.05 mg/ml enrofloxacin in the drinking water, or co-treatment of 0.05 mg/ml enrofloxacin and vitamin A in female mice (n = 3–4) on control or VAD diet, as assessed by LC-MS/MS. Data represent mean ± SEM. Significance between antibiotic levels in antibiotic-treated and co-treated mice was determined with a Mann-Whitney test (*, p<0.05; ns, not significant). (TIF) [file pntd.0008737.s008.tif]

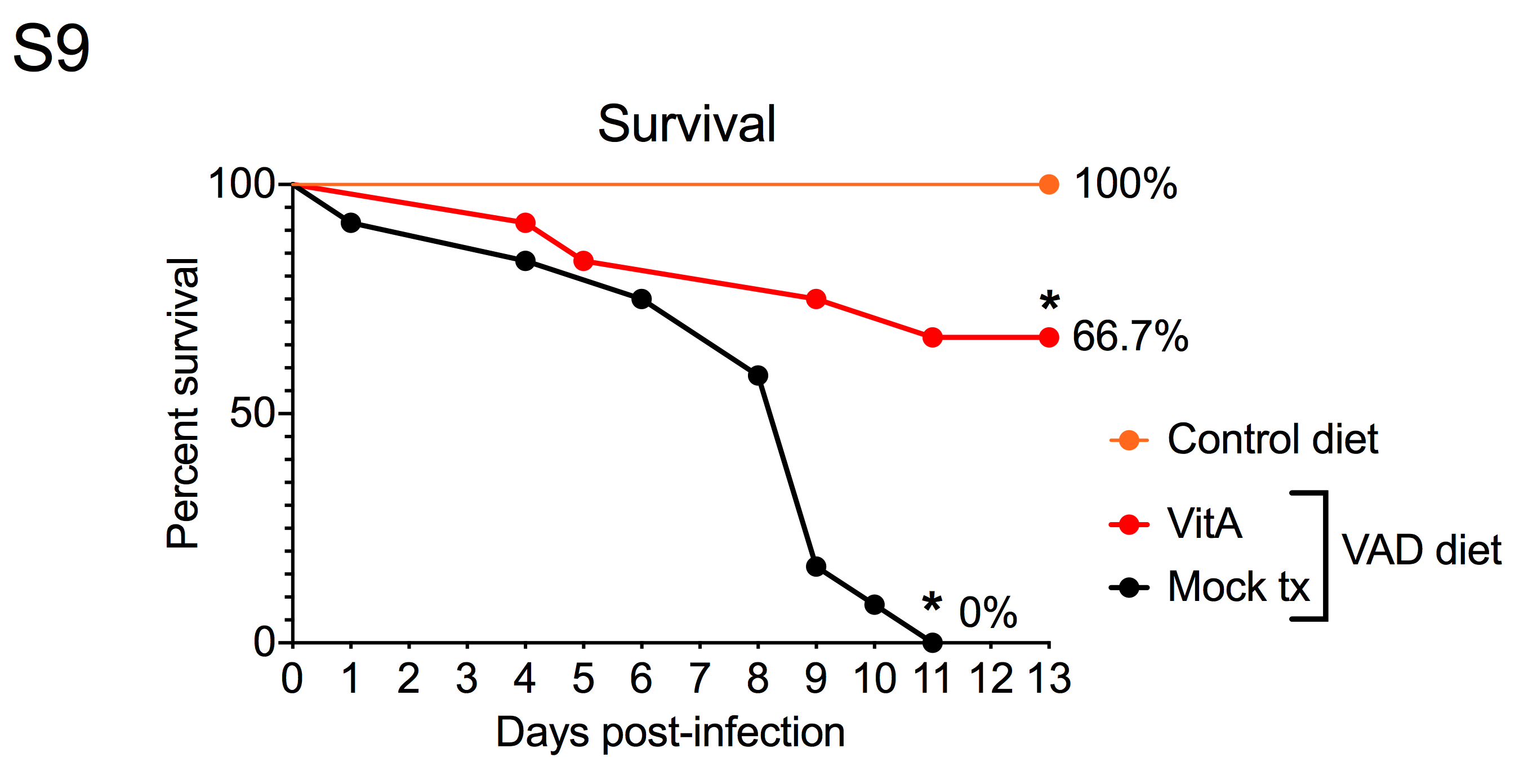

Supplement: S9 Fig — Survival of grouped male and female mice treated with two consecutive doses of either PBS or retinyl palmitate (600 IU delivered by oral gavage) starting 1 day after S. Typhimurium infection. Additionally, VAD mice treated with retinyl palmitate were placed on a control diet replete with vitamin A one-day after S. Typhimurium infection. Data represent percent survival of 12 mice per group from three independent experiments. Pairwise comparisons between the control diet group and each other group was determined with a survival analysis log-rank (Mantel-Cox) test (*, p<0.05). (TIF) [file pntd.0008737.s009.tif]
